# The Use of Blood-Based Biomarkers in the Prediction of Colorectal Neoplasia at the Time of Primary Screening Colonoscopy Among Average-Risk Patients: A Systematic Literature Review

**DOI:** 10.3390/cancers16223824

**Published:** 2024-11-14

**Authors:** R. Liam Sutherland, Dylan E. O’Sullivan, Yibing Ruan, Kristian Chow, Brittany Mah, Dayoung Kim, Robert B. Basmadjian, Nauzer Forbes, Winson Y. Cheung, Robert J. Hilsden, Darren R. Brenner

**Affiliations:** 1 Department of Community Health Sciences, University of Calgary, Calgary, AB T2N 1N4, Canada; robert.sutherland1@ucalgary.ca (R.L.S.); robert.basmadjian@ucalgary.ca (R.B.B.); nauzer.forbes@ucalgary.ca (N.F.); winson.cheung@albertahealthservices.ca (W.Y.C.); rhilsden@ucalgary.ca (R.J.H.); 2 Forzani and MacPhail Colorectal Cancer Screening Centre, Alberta Health Services, Calgary, AB T2N 1N4, Canada; dylan.osullivan@ucalgary.ca (D.E.O.); yibing.ruan@albertahealthservices.ca (Y.R.); kristian.chow@ucalgary.ca (K.C.); brittany.mah@ucalgary.ca (B.M.); dayoung.kim@ucalgary.ca (D.K.); 3 Cancer Epidemiology and Prevention Research, Alberta Health Services, Calgary, AB T2N 1N4, Canada; 4 Department of Oncology, University of Calgary, Calgary, AB T2N 1N4, Canada; 5 Department of Medicine, University of Calgary, Calgary, AB T2N 1N4, Canada

**Keywords:** screening, colorectal cancer, risk prediction, biomarkers, blood, colonoscopy

## Abstract

Background/Objectives: Risk prediction models (RPMs) for colorectal cancer (CRC) could facilitate risk-based screening. Models incorporating biomarkers may improve the utility of current RPMs. We performed a systematic review of studies reporting RPMs for CRC that evaluated the impact of blood-based biomarkers on clinical outcome prediction at the time of screening colonoscopy in average-risk populations. Methods: We conducted a search of MEDLINE, Web of Science, and PubMed databases from inception through April 2024. Studies that developed or validated a model to predict risk of CRC or its precursors were included. Studies were limited to those including patients undergoing average-risk CRC screening. Results: Sixteen studies published between 2015 and 2024 were included. Outcomes included CRC (16 studies) and high-risk adenomas (1 study). Using a complete blood count was the most common biomarker and was able to achieve an AUC of 0.82 and a specificity of 0.88. Other blood-based biomarkers included were various serum proteins/metabolites/enzymes, plasma metabolites, insulin-related factors, and anemia markers. The highest-performing model, with an AUC of 0.99, involved the use of a plasma metabolite panel. Conclusions: The evidence base of RPMs for CRC screening is expanding and incorporating biomarkers, which remain a prominent aspect of model discovery. Most RPMs included a lack of internal/external validation or discussion as to how the model could be implemented clinically. As biomarkers improve the discriminatory potential of RPMs, more research is needed for the evaluation and implementation of RPMs within existing CRC screening frameworks.

## Simple Summary

This study looked at different ways to predict the risk of colorectal cancer using blood tests. Sixteen studies from 2015 to 2024 were reviewed, focusing on people with an average risk of cancer who were undergoing a colonoscopy for screening. The most common test was a complete blood count, which showed good accuracy. Other tests included tests of proteins, metabolites, enzymes, and markers related to insulin and anemia. The best results came from a test using a group of plasma metabolites. While these tests could make cancer screening more accurate, many studies did not show how these models could be used in real-life settings. More research is needed to understand how these models can be effectively used in current screening programs.

## 1. Introduction

CRC is the third most common cancer diagnosed and the second leading cause of cancer-related deaths worldwide [1]. Despite the existence of population-based screening programs in many Western countries, the burden of CRC remains substantial, where 9% of all estimated cancer-related deaths can be attributed to CRC [1]. Traditional risk factors, such as family history, age, and lifestyle factors, have limitations in accurately predicting individual susceptibility [2,3,4,5]. While colonoscopy is currently recommended as a first-line CRC screening modality, barriers to uptake include the financial resources required at the systems level and invasiveness and discomfort at the patient level [6]. Currently, risk assessment for CRC screening is based solely on established comorbid conditions that are known to increase one’s risk of CRC, including but not limited to irritable bowel disease and family history [7,8]. While lifestyle factors are important when considering one’s risk, they are not included in current clinical risk assessment. Combined with the current lack of established criteria for colonoscopy patient selection beyond family history and stool testing, alternate methods for primary CRC screening are needed [9]. Risk prediction models (RPMs) can aid in CRC screening by providing a means to identify people at a higher risk of developing lesions to select those most likely to benefit from colonoscopy.

While biomarkers have been shown to aid in the prognostication and treatment of CRC, their clinical utility in CRC screening strategies has yet to be established [10]. Biomarkers in general have a broad definition as clinical results that are objectively measured and evaluated as indicators of normal biological processes, pathogenic processes, or pharmacologic responses to a therapeutic intervention [11]. Currently there is only one test that incorporates biomarkers outside of standard fecal testing into CRC screening practices [10,12]. In the United States, Cologuard uses a multitarget stool DNA test that detects both abnormal DNA and blood as a first line of CRC screening [13]. Further, in March of 2024 a new study was published that aimed to develop and test a next-generation multitarget stool DNA test that could display a higher specificity than that of Cologuard [14]. Ultimately, the study’s new test displayed a higher sensitivity for CRC compared to a fecal immunochemical test (FIT), similar to Cologuard, while it had a lower specificity compared to FIT [14]. While there are no official RPMs with blood-based biomarkers used for CRC screening, it is important to note that it is common to use a complete blood count (CBC), or specifically hemoglobin, and ferritin to identify patients with iron-deficiency anemia who may have clinically asymptomatic CRC [15].

Blood-based biomarkers present a potential means to aid in risk triage of screening patients, as in some cases, laboratory values for specific blood-based biomarkers may already exist within patient charts, rendering additional testing unnecessary. Risk stratification for screening may further be improved by finding new blood-based biomarkers that can aid in the identification of patients at a higher risk for developing CRC, thus prioritizing them for primary screening methods such as colonoscopy.

There have been previously published systematic literature reviews regarding the use of risk prediction models for colorectal cancer screening [2,3,4,5]. These reviews have focused on either colorectal cancer or high-risk adenomas among average-risk populations [2,3,4,5]. This review specifically aimed to identify studies that developed risk prediction models examined for intervention at the time of primary screening colonoscopy among average-risk patients. We focus on non-genetic, modifiable biomarkers, as these are central to our research aims. Genetic studies were excluded, as they typically analyze inherited factors that are not modifiable and are not routinely measured in most clinical settings or laboratory workflows. This distinction allows us to concentrate on how blood-based biomarkers could enhance clinical decision making and improve risk prediction models.

## 2. Materials and Methods

We conducted a systematic review adherent to the PRISMA guidelines to examine all risk prediction models for colorectal neoplasia at the time of screening-related colonoscopy [16]. Further, models were only included if they incorporated a blood-based biomarker and were generalizable to the average-risk CRC screening population.

### 2.1. Search Strategy

An electronic search of the Medline, Web of Science, and PubMed databases from inception through April 2024 was performed with no language limits using a combination of subject headings incorporating “colorectal cancer,” “risk/risk factor/risk assessment/chance,” “prediction/model/score”, and “biological marker/biomarker/molecular”. The unabridged search strategy is provided in Supplementary Figure S1. The reference lists of all included manuscripts and relevant reviews were also manually screened but did not yield any studies outside the initial search.

### 2.2. Study Selection

Studies were included if they fulfilled all the following criteria: they developed or evaluated a risk prediction model on an average-risk CRC screening-eligible population free from clinical symptoms of CRC; they developed or evaluated a risk prediction model that incorporated a blood-based biomarker; they used either CRC or high-risk adenomas (HRAs) as the outcome of risk prediction; and any study design was included if it fulfilled the previous criteria. Average risk in this context is widely defined since it is jurisdiction-specific and contains subtle variations. For example, the United States includes those aged 45–74, whereas in Canada the age range is 50–74, and thus studies were included if they were developed on patients aged 40–80 years, free from current and previous history of CRC, genetic conditions such as Lynch syndrome or FAP, or irritable bowel diseases such as Crohn’s or colitis. Studies including only highly selected groups, for example immunosuppressed patients, organ transplant recipients, or those with a family history or personal history of colon and/or rectal cancer, were excluded. General population-based studies outside of populations undergoing screening were excluded to ensure that our review focused on risk prediction models specifically relevant to average-risk individuals undergoing screening colonoscopy. Additionally, genome-wide association studies (GWASs) and genetic-based studies were excluded because they typically analyze inherited genetic factors rather than the modifiable, non-genetic biomarkers that are central to our research aims. Furthermore, studies aimed solely at biomarker associations and discovery without a discernible RPM were not included as they do not evaluate clinical utility and decision making in the context of colorectal cancer screening. By narrowing our scope in this manner, we aim to enhance the relevance and applicability of our findings for effective risk stratification and screening interventions.

Two reviewers independently assessed each abstract, and two reviewers independently assessed all full-text manuscripts (KC, BM, DK, RB). Those deemed not to meet inclusion criteria by both researchers were excluded. Any disagreements were resolved by the primary author (RLS). All relevant information from the included studies was extracted by the primary author (RLS) using a standardized extraction sheet. Study data extracted included the study’s case definition (CRC, HRA, etc.), as well as the methods used for model development (regression, machine learning, etc.), validation techniques, the specific outcome measured, and the quantitative measures of model utility, including the area under the receiver operating characteristic curve (AUC) and sensitivity/specificity.

## 3. Results

### 3.1. Study Details

**Figure 1:**
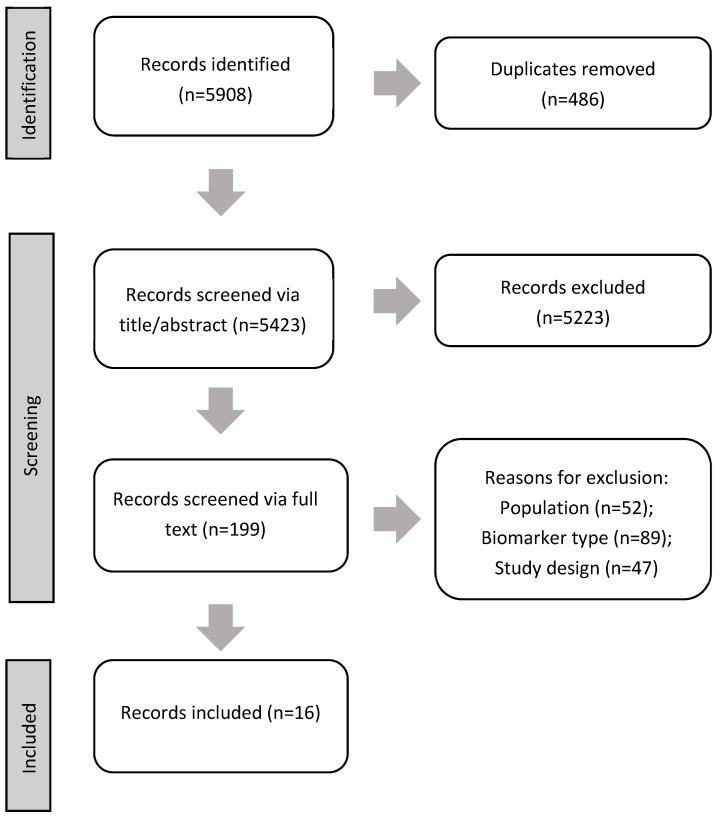
PRISMA flow diagram to represent the flow of studies searched, screened, and included/excluded.

After duplicate removal, 5131 studies were identified. Abstract and title screening was completed in duplicate and yielded 200 studies for full-text screening. Full-text screening yielded 16 studies for inclusion in this review [17,18,19,20,21,22,23,24,25,26,27,28,29,30,31,32]. Figure 1 shows the PRIMSA diagram [33]. Table 1 provides a summary of all included models. Overall, reporting was inconsistent across the included studies. None of the included studies mentioned the use of any reporting standards, including the Transparent Reporting of a multivariable prediction model for Individual Prognosis or Diagnosis (TRIPOD) statement [34]. Further information regarding the risk of bias for the included studies can be found in Supplementary Table S1. Of the 16 studies, all models used colorectal cancer as a prediction outcome; 1 incorporated a second model with advanced adenomas as the outcome [23], and was also able to provide risk estimates for non-advanced adenoma outcomes. Six of the included studies were based on machine learning methods (gradient boosting and random forest methods) [19,22,23,26,28,29] whereas the remaining nine studies used traditional regression modelling techniques. Seven of the included studies featured purely model development methods [20,21,24,25,27,31], four studies specifically mentioned methods regarding internal validation [17,18,29,30], and six studies were able to conduct an external validation of an RPM [19,22,23,26,28,29]. The most common biomarker incorporated was a single-measurement CBC; however, anemia/red blood cell count, plasma metabolite panels, insulin growth factor, serum placenta growth factor, and other various serum protein levels were included in the RPMs. Sample sizes of the included studies ranged from 200 to over 2.8 million patients. Regarding geography, the study locations ranged and included China, the USA, Taiwan, Germany, Canada, the United Kingdom, Spain, Japan, Israel, and South Korea.

### 3.2. Model Performance

Table 2 summarizes the performance (when provided) of all models with CRC as the prediction outcome. The AUCs ranged from 0.66 to 0.99, sensitivity ranged from 31% to 99%, and specificity ranged from 82% to 94%. There were six studies that used a CBC as the incorporated biomarker, and all reported reasonable performance measures with AUCs ranging from 0.776 to 0.82 [19,22,23,26,28,29]. The model with the highest-reported AUC of 0.99 incorporated a plasma metabolite panel to predict CRC and reported sensitivity and specificity of 99.3% and 93.8%, respectively [24]. Finally, while there were some studies that only incorporated specific components of a CBC, they were still able to perform moderately well, with AUCs above 0.75 [17,25,30].

## 4. Discussion

In this study, we reviewed the evidence base for risk prediction models developed at the time of primary screening colonoscopy among average-risk participants. Our focus was on models that incorporated at least one blood-based biomarker, as these biomarkers offer insights into an individual’s current health status and potential CRC risk. By consolidating the existing literature, we aim to demonstrate how the integration of blood-based biomarkers into risk assessment models could enhance individualized risk prediction, facilitating more accurate stratification of patients based on modifiable factors. This approach not only has the potential to improve clinical decision making but also optimizes resource allocation for preventive strategies such as colonoscopy, ultimately contributing to better outcomes in colorectal cancer screening [35].

This review identified several blood-based biomarkers that have been investigated in the prediction of both CRC and HRAs. Overall, there was a general lack of consistency with reporting performance characteristics for published RPMs. Despite only one study being conducted prior to their publication, there were no studies that reported the use of the TRIPOD guidelines [34]. While most reported at minimum the AUC, it was often difficult to determine if the result was the internally validated AUC or the AUC from the original model, where ideally both would be reported. Further, while the AUC can be a helpful metric for model fit, certain performance characteristics such as the sensitivity and specificity of the model were often unreported.

Among the studies included in this review, the incorporation of a single-time CBC measurement into risk prediction was examined the most [19,23,26,29]. Two external validation studies were included involving CBC biomarkers and identified that CBC information can be a valuable addition to risk prediction modelling in a CRC screening context [23,26]. In 2016, Kinar and colleagues published a study on the development and external validation of an RPM that incorporated age, sex, and a CBC (including red blood cells, white blood cells, hemoglobin, and hematocrit) that was subsequently named ColonFlag [29]. Their model was then externally validated, and ultimately displayed an AUC of 0.82 and specificity of 88%. Since its publication, two other studies have been published that aimed to externally validate ColonFlag in either a different population, or with a different outcome relevant to CRC screening [23,26]. In 2017, Hornbrook et al. aimed to validate the ColonFlag model in a US community-based insured adult population [26]. Then, Birks and colleagues published an external validation aiming to predict CRC within 18–24 months compared to Kinar’s original 3–6-month analysis [28,29]. Further, Hilsden and colleagues published a study in 2018 that aimed to understand if ColonFlag could also predict the presence of high-risk adenomatous polyps, a well-defined precursor to CRC [23]. Then, in 2019 Ayling validated ColonFlag for use on anemic patients, and finally in 2020 Schneider performed an external validation using a racially and ethnically diverse database with over 2.8 million patients [19,22]. These studies were some of the first to demonstrate how the use of blood-based biomarkers could assist physicians in identifying patients at higher risk of developing CRC, and highlighted the ease, low cost, and flexibility of using simple CBCs to improve screening efficiency.

However, challenges remain in translating these findings into clinical practice. One challenge is the need for further validation and standardization of biomarkers across different populations [35]. While there have been external validation studies for ColonFlag, more studies into different patient subgroups and clinical settings are essential to account for subtle population differences, such as demographic makeup, within the CRC screening population. This helps to ensure the generalizability and accuracy of risk prediction models. Another challenge lies in integrating biomarkers into existing risk assessment models and screening programs [35]. The development of comprehensive risk prediction models that incorporate biomarkers alongside established risk factors is a priority. Such models should first consider the biomarker’s clinical utility and then investigate its cost-effectiveness and feasibility, and those of incorporating biomarkers into routine screening protocols [36]. Once an RPM has been appropriately developed and validated both internally and externally, it is imperative that trials be conducted alongside current screening methods to evaluate the possible effectiveness of the RPM in a clinical setting. In 2022, a systematic review was published that identified seven studies regarding the economic evidence of risk-tailored CRC screening strategies [36]. Four of the included studies concluded that risk-tailored screening would be considered cost-effective [37,38,39,40]. In contrast, Ladabaum et al. published a clinical and economic impact study that concluded that given the state of current research, uniform screening is likely to be preferred over tailored screening if the risk prediction tool is associated with even minor misclassification issues [41]. Overall cost-effectiveness is directly impacted by the discrimination of the prediction tool, the accuracy of the technique, and the uptake rate to screening [36]. To date, there is only one randomized controlled trial that has been published comparing traditional colonoscopy-based screening, FIT, and the use of personalized risk scoring (www.chictr.org.cn, Identifier: ChiCTR1800015506 accessed on 28 August 2024) [42,43]. This trial not only examined how screening outcomes differed, but also noted the participation rates and subsequent resource utilization. Ultimately, pragmatic trials like these are paramount in understanding how effective RPMs can be in a screening setting, and while many models still require proper validation, the examination of model implementation should always be considered.

Cost-effectiveness will be a pivotal consideration moving forward; biomarker tests must demonstrate not only improved clinical outcomes but also offer practical financial solutions for healthcare systems and patients. Balancing personalized screening benefits with affordability and access will be critical in future research and implementation efforts.

It is important to acknowledge the limitations of our review. The aim of this review was to identify risk prediction models specifically developed for use at the time of screening colonoscopy using blood-based biomarkers. GWASs are often based on blood testing, but they were not included because they are generally conducted on large cross-sectional datasets that often do not specify a specific population. These studies often function to identify specific genes of interest in CRC risk and were therefore excluded from this review. In this review, we did not identify large evidence bases that could be combined or evaluated within sets of predictor variables and incorporated biomarkers. Given this limitation in the evidence base and a lack of consistent model performance, a meta-analysis was not possible. Additionally, RPMs are influenced by various factors, such as study design, sample size, and included risk factors. It is important to understand the role of all relevant predictors when it comes to using blood-based biomarkers in the prediction of CRC. Understanding that the risk profile for CRC is varied and often a result of a combination of many comorbidities and lifestyle factors is paramount to ensuring risk stratification. Improper consideration of these factors may introduce biases and affect the generalizability of the findings. Due to the considerable heterogeneity of the included studies, as previously mentioned, and the fact that some models were developed using traditional logistic regression while others utilized machine learning techniques, direct comparisons should be approached with extreme caution given the differences between these methods. However, evaluating and describing the work that has been conducted in this field is still important, both to fully understand how research in risk prediction modelling for CRC screening is progressing, and to ensure future research can be conducted in a manner that is transparent enough to enable more direct comparison in the future.

## 5. Conclusions

In conclusion, biomarkers for colorectal cancer (CRC) risk prediction have the potential to enhance screening approaches for CRC. This review adds to the growing evidence base by highlighting the promise of blood-based biomarkers in optimizing CRC screening. However, to translate these findings into routine clinical practice, further research, validation, and standardized reporting are essential. Although the current literature indicates the potential of these biomarkers, none have been comprehensively assessed for integration into existing screening protocols.

Future studies should work to validate across relevant populations and healthcare settings. Testing biomarkers in varied screening settings, including rural and resource-limited areas, may help to maximize their utility. Integrating biomarker testing with existing methods, such as colonoscopies and stool-based tests, offers the potential to enhance diagnostic accuracy by reducing false positives and negatives. However, this integration must occur seamlessly within routine clinical workflows to avoid added complexity and cost. Ultimately, while significant gaps remain for risk prediction models in CRC screening, the incorporation of biomarkers could facilitate more personalized approaches to screening and intervention to reduce the impact of CRC.

## Figures and Tables

**Table 1:** Summary of included risk prediction models that include blood-based, non-genetic biomarkers at the time of screening colonoscopy.

Author, Year	Country	Outcome *	Method **	Biomarker	Study Type
Zhang, 2024 [17]	China	CRC	LR	Red blood cell count, anemia, and platelet count, and red cell distribution width—standard deviation	Development and validation
Fang, 2021 [18]	USA	CRC	LR	25-hydroxyvitamin D, total adiponection, C-reactive protein, growth/differentiation factor 15, insulin-like growth factor 1, insulin-like growth factor-binding protein, interleukin 6, leptin receptor, sex hormone binding globulin, and tumour necrosis factor receptor superfamily member 1B	Development and validation
Schneider, 2020 [19]	USA	CRC	ML (gradient boosting/random forest)	Complete blood count	External validation
Ayling, 2019 [22]	UK	CRC	ML (gradient boosting/random forest)	Complete blood count	External validation
Wei, 2019 [20]	Taiwan	CRC	LR	Serum placenta growth factor	Development
Bhardwaj, 2019 [21]	Germany	CRC	LR	Mannan binding lectin serine protease 1, serum paraoxonase lactinase 3, transferrin receptor protein 1, and amphiregulin	Development
Hilsden, 2018 [23]	Canada	CRC, AA, non-AA	ML (gradient boosting/random forest)	Complete blood count	External validation
Birks, 2017 [28]	UK	CRC	ML (gradient boosting/random forest)	Complete blood count	Development and external validation
Goshen, 2017 for males [27]	Israel	CRC	LR	Hemoglobin, mean corpuscular volume, monocyte count, platelets, alkaline phosphatase, alanine aminotransferase, aspartate aminotransferase, iron, and ferritin	Development
Goshen, 2017 for females [27]	Israel	CRC	LR	Hemoglobin, mean corpuscular volume, neutrophil count, platelets, red blood cell distribution width, alanine aminotransferase, protein, iron, and ferritin	Development
Hornbrook, 2017 [26]	USA	CRC	ML (gradient boosting/random forest)	Complete blood count	External validation
Navarro-Rodriguez, 2017 [25]	Spain	CRC	LR	Fibrinogen, hemoglobin, relative neutrophil, absolute platelet count, and eosinophils	Development
Nishiumi, 2017 [24]	Japan	CRC	LR	Pyruvic acid-meto-TMS, glycolic acid-2TMS, tryptophan-3TMS, palimtoleic acid-TMS, fumaric acid-2TMS, ornithine-4TMS, lysine-4TMS, and 3-hydroxyisovaleric acid-2TMS	Development
Yang, 2017 [32]	South Korea	ACN	LR	Fasting glucose, low-density lipoprotein cholesterol, and carcinoembryonic antigen	Development and validation
Boursi, 2016 [30]	UK	CRC	LR	Hematocrit, mean corpuscular volume, lymphocyte count, and neutrophil–lymphocyte ratio	Development and validation
Kinar, 2016 [29]	Israel and UK	CRC	ML (gradient boosting/random forest)	Complete blood count	Development, validation, and external validation
Pankaj, 2015 [31]	South Korea	CRC	LR	IGF-1, IGFBP-3, and C-Peptide	Development

* AA: advanced adenoma; CRC: colorectal cancer; non-AA: non-advanced adenoma. ** LR: logistic regression; ML: machine learning.

**Table 2:** Risk prediction model area-under-the-curve, sensitivity, and specificity summary statistics from the models included within this review when available.

Author, Year	Biomarker	AUC *	Sensitivity (%) *	Specificity (%) *
Fang, 2021 [18]	Circulating plasma panel	0.73, men; 0.66, women		
Schneider, 2020 [19]	Complete blood count	0.78	35.4	
Bhardwaj, 2019 [21]				
Wei, 2019 [20]	Serum placenta growth factor	0.797	0.5708	0.8614
Hilsden, 2018 [23]	Hemoglobin, WBC, and platelets			
Hornbrook, 2017 [26]	Complete blood count	0.81		
Navarro Rodriguez, 2017 [25]	Fibrinogen, hemoglobin, relative neutrophils, absolute platelet count, and eosinophils	0.854		
Nishiumi, 2017 [24]	Plasma metabolite panel	0.996	99.3	93.8
Kinar, 2016 [29]	Complete blood count	0.82		88

* Significant figures were included as reported in the original manuscripts.

## Supplementary Materials

The following supporting information can be downloaded at https://www.mdpi.com/article/10.3390/cancers16223824/s1: Figure S1: Search strategy; Table S1: Risk of bias for included studies.
