# Supplementary material for: The Use of Blood-Based Biomarkers in the Prediction of Colorectal Neoplasia at the Time of Primary Screening Colonoscopy Among Average-Risk Patients: A Systematic Literature Review
Source: Cancers (Basel). 2024 Nov 14;16(22):3824. doi: 10.3390/cancers16223824 (PMC11593258; doi:10.3390/cancers16223824)
Supplement: Supplementary file 1 [file cancers-16-03824-s001.zip › cancers-3231703-supplementary.pdf]

Supplemental Files

# ▲	Searches
1	exp Risk/ or exp Risk Assessment/
2	(risk* or "risk adjust*" or "risk* strat*" or strat* or adjust*).mp.
3	exp "Early Detection of Cancer"/
4	exp Mass Screening/
5	(screen* or "screen* program* guide*" or "Screen* guide*").mp.
6	exp colorectal neoplasms/ or exp adenomatous polyposis coli/ or exp colonic neoplasms/ or exp rectal neoplasms/
7	("colon tumo?r" or "colon dysplasia*" or "colon malign*" or "colon canc*" or "rect* tumo?r" or "rect* dysplasia*" or "rect* malign* dysplasia*" or "colorectal malign*" or "colorect* canc*" or "gastro* canc*" or CRC).mp.
8	1 or 2
9	3 or 4 or 5
10	6 or 7
11	8 and 9 and 10
12	limit 11 to animals
13	limit 11 to (animals and humans)
14	12 not 13
15	11 not 14
16	limit 15 to (case reports or comment or editorial or letter or review or congress)
17	15 not 16

Supplemental Figure S1: Search strategy used for systematic review.

Supplemental Table S1: Risk of Bias assessment using the PROBAST Tool[33].

Study	ROB				Overall
	Participants	Predictors	Outcome	Analysis	ROB
Zhang, 2024	+	-	+	+	-
Fang, 2021	+	+	+	-	-
Schneider, 2020	-	+	+	+	-
Ayling, 2019	+	-	+	-	-
Wei, 2019	+	+	+	+	+
Bhardwaj, 2019	+	-	+	+	-
Hilsden, 2018	+	+	+	+	+
Birks, 2017	+	-	+	-	-
Goshen, 2017	-	-	+	-	-
Hornbrook, 2017	+	-	+	+	+
Navarro-Rodriguez, 2017	-	+	+	-	-
Nishiumi, 2017	+	-	-	+	-
Yang, 2017	+	-	+	-	-
Boursi, 2016	-	+	+	+	-
Kinar, 2016	+	-	-	+	-
Pankaj, 2015	+	+	+	+	+
